# Sentential inference bridging between lexical/grammatical knowledge and text comprehension among native Chinese speakers learning Japanese

**DOI:** 10.1371/journal.pone.0284331

**Published:** 2023-04-13

**Authors:** Katsuo Tamaoka, Hiromu Sakai, Yayoi Miyaoka, Hajime Ono, Michiko Fukuda, Yuxin Wu, Rinus G. Verdonschot

**Affiliations:** 1 Hunan University, Changsha, China; 2 Nagoya University, Nagoya, Japan; 3 Waseda University, Shinjuku City, Japan; 4 Hiroshima University of Economics, Hiroshima, Japan; 5 Tsuda University, Kodaira, Japan; 6 Bunkyo University, Koshigaya, Japan; 7 Xi’an International Studies University, Xi’an, China; 8 Max Planck Institute for Psycholinguistics in Nijmegen, Nijmegen, The Netherlands; The University of Auckland, NEW ZEALAND

## Abstract

The current study explored the role of sentential inference in connecting lexical/grammatical knowledge and overall text comprehension in foreign language learning. Using structural equation modeling (SEM), causal relationships were examined between four latent variables: lexical knowledge, grammatical knowledge, sentential inference, and text comprehension. The study analyzed 281 Chinese university students learning Japanese as a second language and compared two causal models: (1) the *partially-mediated model*, which suggests that lexical knowledge, grammatical knowledge, and sentential inference concurrently influence text comprehension, and (2) the *wholly-mediated model*, which posits that both lexical and grammatical knowledge impact sentential inference, which then further affects text comprehension. The SEM comparison analysis supported the wholly-mediated model, showing sequential causal relationships from lexical knowledge to sentential inference and then to text comprehension, without significant contribution from grammatical knowledge. The results indicate that sentential inference serves as a crucial bridge between lexical knowledge and text comprehension.

## Introduction

In most written or spoken texts, a specific message is conveyed through a series of semantically related sentences that form meaningful connections. To understand these texts, one must grasp the meaning of the individual words [1–4] and have proficiency in the grammatical rules that prescribe word arrangement within sentences [5–9]. As a result, a combination of lexical and grammatical knowledge is essential for effective sentence comprehension.

However, there is a significant difference between understanding a single sentence and comprehending the overall message conveyed by a text. A text typically consists of multiple sentences, and the comprehension of its message relies on the rules of inference that connect one sentence to another. Sentential inference is thought to facilitate the semantic connections between previously introduced and new sentence information [10–14], ultimately leading to the comprehension of a text’s complete message [15–16].

The current study aims to investigate the role of sentential inference as a mediator between lexical and grammatical knowledge and overall text comprehension.

## Background

### The role of lexical knowledge during text comprehension

The role of lexical knowledge in text comprehension has been extensively studied in first and second language acquisition. Research on language acquisition in native English-speaking children [1, 17–19] has demonstrated that lexical knowledge is a significant contributor to text understanding and reading comprehension. LaBerge and Samuels [2] introduced the concept of *‘automaticity*,’ which suggests that proficient readers can process words in a text effortlessly. They suggested this ultimately allows children to focus their cognitive efforts on higher-order comprehension skills once they have mastered automatic word processing.

Beck, Perfetti, and McKeown [1] examined the connection between word processing and reading comprehension as well. Over a five-month period, they trained fourth-grade students to establish semantic networks, leading to improved word processing efficiency and enhanced reading comprehension compared to a control group. Similarly, using Cantonese, Leong and Ho [20] found that lexical knowledge significantly influenced Chinese reading comprehension in a study involving 361 Chinese secondary school students.

In a study of Japanese language acquisition, Tamaoka, Leong & Hatta [4] found that word processing speed, an indicator of automaticity, greatly influenced reading comprehension. They observed significant differences in processing alphabetic loanwords (*katakana*) and Chinese-origin words (*kanji*) between skilled and less-skilled readers from grades four to six. Skilled readers were better at deciphering unknown pronunciations of difficult kanji characters using phonetic cues. This highlights the importance of phonological and orthographic morphemes in reading comprehension (see also [21]).

Similarly, Takahashi [3] determined that lexical knowledge was the most crucial factor in reading comprehension for Japanese-speaking children in grade five. In a subsequent longitudinal study [22], Japanese-speaking children from grades one to five were studied and it was discovered that three primary factors—word pronunciation speed, reading memory span, and lexical knowledge—significantly impacted reading comprehension in younger children (grades one to three). However, upon reaching grade five, lexical knowledge emerged as the sole influential factor, with word pronunciation speed and reading memory span no longer having a significant effect on reading comprehension. Takahashi [22] attributed this change to the increasing complexity of reading contexts and the expanding range of vocabulary used in texts as children progress through the grades.

So far, our primary focus has been on the text comprehension of monolinguals. Nevertheless, it seems necessary to point out that also for bilinguals, strategies to text comprehension might overlap and warrant an investigation. In research exploring the acquisition of English as a second language by native Chinese speakers, Zhang [23] employed Structural Equation Modeling (SEM) to investigate the contributions of lexical and grammatical knowledge to reading comprehension. Zhang’s findings revealed that lexical knowledge had a significant impact on reading comprehension, whereas grammatical knowledge offered a more modest contribution.

Considering the significant role lexical knowledge seems to play in reading comprehension, some have introduced the concept of a ‘*lexical threshold*’ to determine the minimum lexical knowledge necessary for accurate text comprehension. Hu and Nation [24], Nation [25], and Stahl and Nagy [26] posited that understanding 98% of the words in a written English text is required to achieve accurate comprehension. In the case of native Chinese speakers learning Japanese, Komori, Mikuni, and Kondo [27] estimated that recognizing 96% of the words in a general written text is essential for comprehension. Note that this number still indicates that about 4% of the vocabulary in a text would be unknown. Additionally, Mikuni et al. [28] calculated the threshold for listening comprehension to be around 93%. As spoken language is typically less lexically dense than written language, it is possible that listening comprehension can be achieved with a lower lexical threshold than reading comprehension. However, it is evident that a high level of lexical knowledge is crucial for attaining text comprehension in both spoken and written Japanese.

In a second language study by Zhang, Zhang, Li, and Zhang [29], factors impacting Chinese reading comprehension were examined by testing 447 English-speaking Chinese learners from college-level study-abroad programs. The study found that morphological awareness and lexical knowledge both influenced reading comprehension through lexical inference. As Chinese texts are typically composed of morphological (logographic) characters called *hànzì*, which do not contain inflections for verbs or adjectives, knowledge of hànzì morphology and compound words is essential to understand the text. Approximately 75–80% of Chinese words consist of two or more hànzì characters [30]. Zhang et al. [29] identified lexical inference as a mediating factor between morphological/lexical knowledge and reading comprehension, indicating a sequential relationship between the understanding of hànzì characters, their compound words, and the lexical relations of multiple words. Consequently, morphological/lexical knowledge and lexical inference seem to play crucial roles in Chinese reading comprehension.

Other research has shown that adult native Chinese speakers learning Japanese also make use of morphological/lexical knowledge when learning English. For example, Koda (2000) examined native Chinese and Korean speakers learning English and found that while both groups showed similar sensitivity to English morphological structure, Chinese learners were more efficient in integrating morphological and contextual information during English sentence processing. This strong contribution of morphological awareness to reading comprehension was also evident among native Chinese children in elementary school who learnt English as a second language. Wang, Cheng, and Chen [31] reported that both Chinese and English morphological awareness contributed to Chinese reading comprehension in these children, referring to this phenomenon as "*cross-language morphological transfer*." Specifically, Native Chinese speakers initially learn to use hànzì morphological awareness for text comprehension in their first language (Chinese). This morphological/lexical approach, which they acquired in Chinese, can then potentially be applied through lexical inference to comprehend English text as well.

Koda’s [32] findings can also be relevant to native English speakers learning Chinese as a second language. For example, Zhang et al. [29] suggested that morphological awareness and lexical knowledge, through lexical inference, significantly contribute to the comprehension of Chinese text. Due to the linguistic characteristics of the Chinese language, native English speakers likely rely on hànzì for morphological awareness and utilize lexical inferences to understand Chinese text. Consequently, the morphological awareness approach is employed in both cases: from Chinese as a first language to English as a second language [32], and from English as a first language to Chinese as a second language [29, 31]. When learning Chinese as a second or foreign language, morphological awareness of hànzì is essential for text comprehension. Note that although there are many inconsistencies in the usage of this terminology, typically, a “foreign” language is not spoken in the region where it is taught whereas a second language is. Therefore, in both directions of language acquisition, *morphological awareness* appears to be a critical factor.

Furthermore, lexical knowledge plays a crucial role in Japanese text comprehension for native Chinese speakers learning Japanese as a foreign language. Chen [33] found that among 4,600 Japanese kanji-compound words, 54.5% had the same characters and meanings as in Mandarin Chinese (e.g., 数学 “mathematics”), while 14.9% shared the same characters but had similar meanings (e.g., 高校 “college” in Chinese; “high school” in Japanese). Only 4.1% of the words had the same characters but different meanings (e.g., 暗算 “mental arithmetic” in Japanese, “to plot” in Chinese). If minor orthographic differences between Chinese hànzì and Japanese kanji characters are disregarded, native Chinese speakers can understand approximately 98.1% of commonly used Japanese kanji before even studying the language [34, 35]. This high degree of similarity explains why native Chinese speakers often rely heavily on kanji-based lexical items to comprehend written Japanese texts. As a result, native Chinese speakers learning Japanese are likely to leverage their knowledge of hànzì to understand words in Japanese text.

Japanese differs from Chinese in that it utilizes three scripts instead of only the orthographic (hànzì) script [36]. Firstly, Japanese text incorporates Chinese-originated words (Kango) written in Chinese morphemic *hànzì* characters (known as *kanji* in Japanese) which predominantly convey conceptual meanings. Additionally, there are two phonetic scripts: *katakana* and *hiragana*. Katakana is primarily used for transcribing alphabetic loanwords, with word etymology differentiated by the use of kanji and katakana. Hiragana, the third script, is commonly employed for grammatical functions, although it has other uses as well (e.g., to write specific native Japanese words, such as: きれい,/kirei/ “beautiful”). Given that lexical and grammatical functions can be visually distinguished to some extent by the three scripts in Japanese, the causal relationship between morphological awareness, lexical knowledge, and lexical inference leading to text comprehension in Chinese [29] may not operate in the same manner for understanding Japanese texts.

### The role of grammatical knowledge for text comprehension

Despite some researchers (e.g., [5–6, 8–9]) highlighting the significance of grammatical knowledge in text comprehension, it has often been overshadowed by lexical knowledge. Weir [9] reported a strong correlation (r = 0.67) between grammatical knowledge and reading comprehension among non-native English speakers studying English at higher education institutions in the UK. In a study using SEM with 588 native-Japanese speaking university students learning English in Japan, Shiotsu and Weir [7] found a strong causal relationship between grammatical knowledge and reading comprehension, whereas lexical knowledge did not show such a relationship. However, the same study also revealed a comparable contribution of lexical and grammatical knowledge to reading comprehension for 107 native Japanese university students studying in the UK.

These observed differences in the contribution of lexical and grammatical knowledge to reading comprehension among Japanese students in Japan and the UK could be attributed to their distinct learning environments. Students in the UK would have more opportunities to use English in daily life and receive instruction from native English speakers, unlike their counterparts in Japan. Consequently, Japanese students in English-speaking countries would encounter various words and expressions regularly, leading to a simultaneous reliance on both lexical and grammatical knowledge for text comprehension.

On the other hand, Japanese students in Japan have limited exposure to English outside the classroom and might adopt a strategy of understanding English by translating it into Japanese. Given the significant differences in grammatical features between the two languages, grammatical knowledge becomes vital for translation. As a result, these students might depend more on grammatical knowledge for text comprehension. The contrasting English learning environments in Japan and the UK could therefore explain the diverging outcomes in the studies.

For native Japanese speakers learning English, Yamashita [37] found that while both lexical and grammatical knowledge had a significant impact on reading comprehension, lexical knowledge seemed to contribute more. Similarly, Bosser [38] found that both lexical and grammatical knowledge were important predictors for Dutch reading comprehension among native Turkish speakers learning Dutch as a second language. However, Bosser [38] also observed that lexical knowledge was a stronger predictor than grammatical knowledge. Therefore, although grammatical knowledge does contribute to reading comprehension, its influence appears to be less significant than that of lexical knowledge.

Yamato, Tamaoka, and Chu [39] studied the impact of lexical and grammatical knowledge on the phrase-by-phrase processing of Japanese texts (i.e., self-paced reading) by native Chinese speakers learning Japanese. To compare reading speeds, participants were divided into three groups based on their Japanese vocabulary and grammar test scores. They found that lexical knowledge had a more significant influence on reading speed than grammatical knowledge, and it contributed to reading speed for both single phrases and continuous sequences of phrases. However, grammatical knowledge’s impact was limited to certain phrases with complex structures, suggesting that grammar still may play a role in resolving structural difficulties.

Yamato and Tamaoka [40] compared 20 matched pairs of native Chinese and Korean speakers, who were equally proficient in lexical and grammar knowledge. The study found that native Chinese speakers processed words in Japanese kanji characters more quickly than Korean speakers. Due to the similarity between Chinese and Japanese kanji script [41], Chinese speakers were able to process kanji-presented words faster. This suggests that Japanese text comprehension may be partially achieved through morphological/lexical knowledge. As Chinese does not have verb and adjective inflections [42, 43], native Chinese speakers might primarily focus on Japanese kanji characters for text comprehension, similar to their approach in Chinese.

### Bridging between lexical/grammatical knowledge and text comprehension

Lexical and grammatical knowledge are essential for understanding text, but there is a distinction between this knowledge and overall text comprehension [44]. We typically use our life experiences to deduce the meaning of partially understood texts. In reading and listening comprehension, we make inferences based on cues provided within sentences. This information is not explicitly stated, so we depend on our experiential knowledge to decipher the text’s meaning. Zhang et al. [29] labeled this process as *lexical inference*, which involves logical reasoning within individual sentences.

To fully comprehend a text’s message, a broader form of inference, called *sentential inference*, is needed to connect different sentences. Kintsch [15] and van Dijk & Kintsch [16] introduced two textual structures: *microstructure* and *macrostructure*. Microstructure concerns lexical and morphosyntax relations within a sentence, while macrostructure deals with inter-sentential relations. Lo et al. [45] found that cognitive-linguistic skills contribute differently to microstructure and macrostructure understanding in Chinese elementary school children. Intra-sentence microstructural comprehension helps in understanding single sentences (lexical inference), while inter-sentence macrostructure comprehension aids in understanding larger texts (sentential inference).

Long et al. [13] discovered that skilled readers outperformed less-skilled readers in identifying theme-related inferences in specific passages. Murray and Burke [14] noted that high-skilled readers automatically recalled related lexical items, while low-skilled readers did not. These first-language understanding studies suggest that the ability to draw lexical and sentential inferences is crucial for reading comprehension.

The current study focused on three aspects of sentential inference, namely: intentional expressions, co-referential resolution, and causal relations. First, understanding indirect messages in a series of sentences, such as intentions not explicitly stated, is necessary to comprehend the actual message. For example, a listener can infer the unspoken intention of a speaker’s comment about a cold room and act accordingly, like closing a nearby window. This ability to grasp indirect speech is important for overall text comprehension.

The second aspect of sentential inference is co-referential resolution, which occurs when multiple expressions refer to the same person or thing. For example, in “John went to the store, and he bought some cheese," the words "John" and "he" are co-referential because they both refer to the same person. Co-references help link sentences and can refer to a whole sentence’s context, such as a Japanese deictic word like *kore* ‘this’, *sore* ‘that’, or *are* ‘that’ (distal). Understanding these references is what is denoted as co-referential resolution [46].

The last aspect is causal relation, which deals with cause and effect in sentences. Causal relations develop a text’s message and are often indicated by conjunctions like ‘because’, ‘so’, ‘hence’, ‘thus’, or adverbs like ‘accordingly’ and ‘consequently’. In Japanese, causal relations are indicated by conjunctions and subordinate conjunctive particles. Learners of Japanese as a foreign language may face difficulties with conjunctive particles like -*kara* and -*node* due to their similarities, possibly leading to pragmalinguistic failure [47]. Understanding a series of causal relations is vital for grasping a text’s overall message, as it involves processing multiple sentences and their logical reasoning steps.

### Two existing models of text understanding

The current study utilizes structural equation modeling (SEM) to examine the relationship between lexical/grammatical knowledge, sentential inference, and text comprehension, specifically focusing on native Chinese speakers learning Japanese. The SEM analysis encompasses four primary variables: lexical knowledge, grammatical knowledge, sentential inference, and text comprehension.

**Fig 1:**
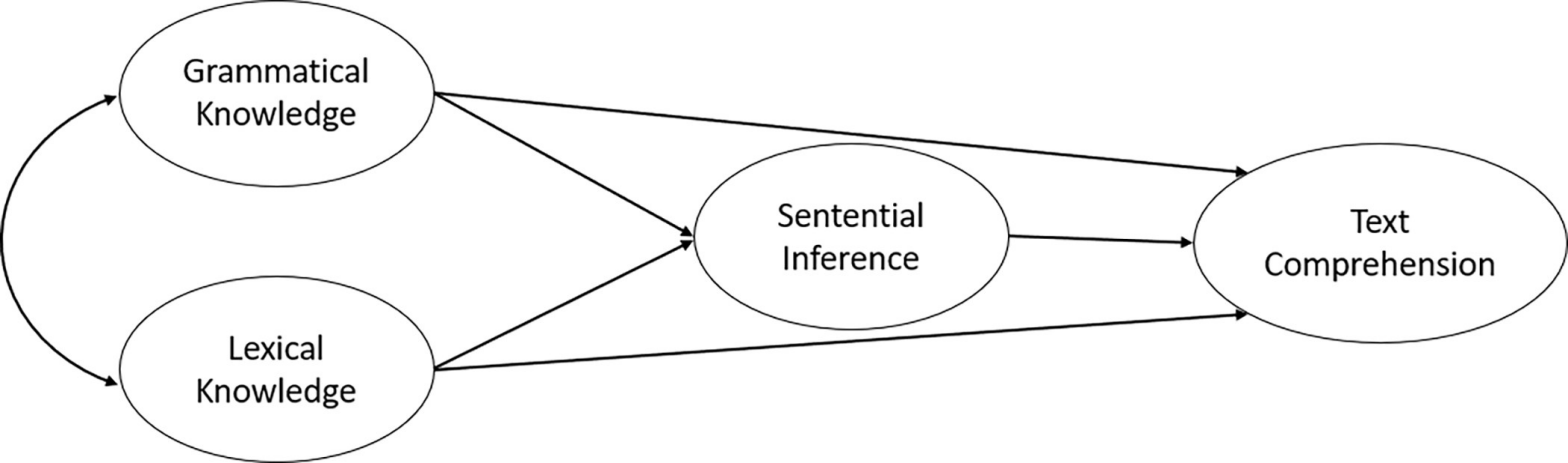
The partially-mediated model of text comprehension.

Two proposed models outline the key factors in text comprehension. The first, the "*partially-mediated*" model, posits that lexical/grammatical knowledge and sentential inference work in tandem to facilitate text understanding (see Fig 1). In this model, second language learners employ lexical and grammatical knowledge to comprehend sentence meaning, while both lexical/grammatical knowledge and sentential inference operate concurrently to aid in understanding the entire text. This model anticipates that word meaning, grammatical features, and their integration with sentential relations all play a role in text comprehension.

**Fig 2:**
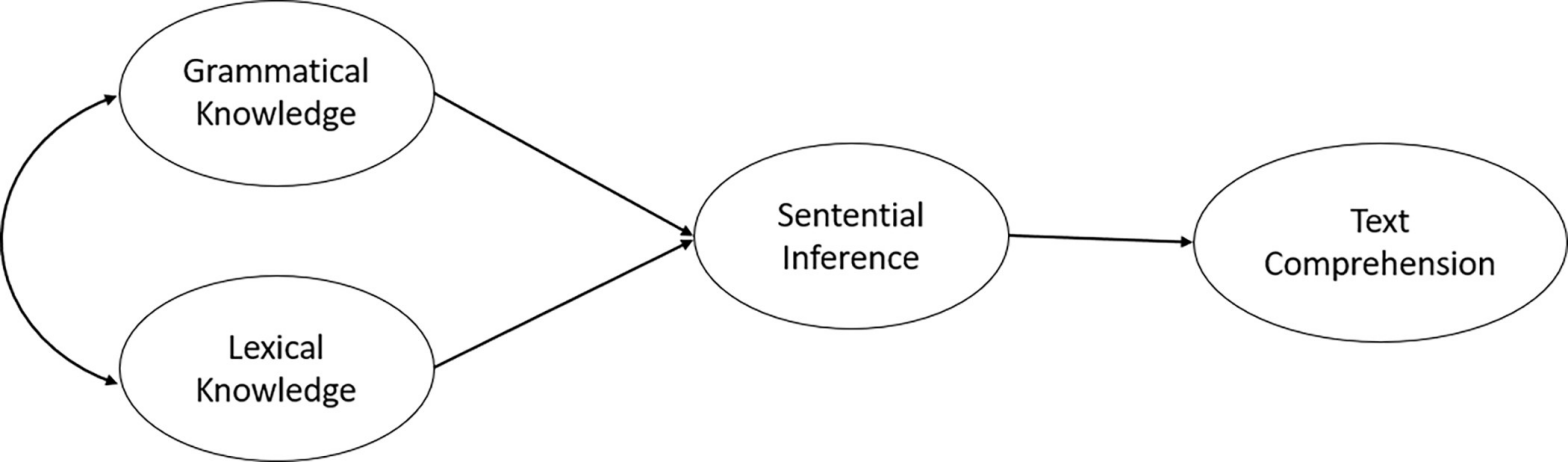
The wholly-mediated model of text comprehension.

The second model, the "*whole-mediated*" model, suggests that lexical and grammatical knowledge offer information to understand individual sentences (see Fig 2). However, comprehending a single sentence does not necessarily convey a text’s overall message. To understand the sequence of messages, sentences must be semantically connected to preceding and following sentences. Consequently, sentential inference may function independently to merge the meanings of multiple sentences, each initially based on lexical/grammatical knowledge. In this model, the final message of a text is understood through the integration of a series of sentential semantic inputs.

Our study tested these two models using a large dataset of native Chinese speakers learning Japanese at Xi’an International Studies University, China. By comparing the models, we investigated the causal relationships between lexical/grammatical knowledge and sentential inference in achieving text comprehension. Notably, since Dixon and Marchman [48] encountered difficulty in determining whether lexical or grammatical knowledge precedes the other, the present SEM analysis incorporated a correlation between them.

## Methodology

### Participants

A total of 281 native Chinese-speaking university students learning Japanese at Xi’an International Studies University participated in this study. They had no prior experience studying Japanese before joining the university. At the time of testing, the students had either one year (N = 146; 124 female) or two years (N = 135; 116 female) of academic Japanese training, and none of them had lived in Japan. Their ages ranged from 16.1 to 26.7 years, with an average age of 19.7 ± 1.0 years for those with one-year of learning experience and 20.8 ± 1.1 years for those with two years of experience. All data collected was securely stored, and participants were given numerical pseudonyms for privacy. Before beginning the study, participants were informed about the study objectives, procedures, risks, and benefits, and verbal informed consent was obtained from each participant to ensure ethical research conduct. The study received approval from the ethics committee of the School of Japanese Culture and Economy at Xi’an International Studies University.

### Measurements

The study investigated four main variables, namely: lexical knowledge, grammatical knowledge, sentential inference, and text comprehension through multiple-choice questions (one correct answer out of four choices), lasting 180 minutes in total (four 45-minute classes).

Lexical knowledge refers to understanding vocabulary in a language, including individual word meanings, spelling, connotations, and how words form phrases and sentences. It is a critical component of language proficiency and communication and is measured in nearly all influential Japanese language proficiency tests (e.g., the Japanese Language Proficiency Test or JLPT). In this study, lexical knowledge was measured through four tests based on word categories: Chinese-origin words (*Kango*), Japanese-origin words (*Wago*), alphabetic loanwords (*Gairaigo*), and function words, which serve a grammatical function rather than conveying lexical meaning. In Japanese, function words are crucial for constructing coherent and meaningful sentences, as they indicate the grammatical relationships between various sentence elements. All words in the test were taken from the JLPT [49]. The Chinese-origin words were selected from two-kanji compound words such as *guti* (‘complaint’), *hukyô* (‘recession’), *syumi* (‘hobby’), *yûbô-da* (‘promising’), *kengaku-suru* (‘to visit’) and *tyûmon-suru* (‘to order’). Examples of Japanese-origin words are *arasuzi* (‘story’), *sakasama* (‘upside-down’), *yayakosii* (‘complicated’), *detarame-na* (‘nonsense’), *hakado-ru* (‘to make progress’), and *unazu-ku* (‘to nod and agree’). All loanwords were taken from alphabetic languages, such as *saizu* (‘size’), *kyaria* (‘career’), *dorai-da* (‘unsentimental’), *rûzu-da* (‘loose’), *massâzi-suru* (‘to massage’) and *suttopu-suru* (‘to stop’). Function words were defined as grammatical words consisting of more than two morphemes with no inflections such as *ga-hayai-ka* (‘no sooner … than …’), *ta-tokoro-de* (‘even if…’), *itaru-made* (‘until…’ or ‘up to…’), *kawa-kiri-ni* (‘start by…’), and *yogi-naku-sa-reru* (‘be obliged to…’). Twelve words in each lexical category, that is: Chinese-origin, Japanese-origin, and alphabetic loanwords consisted of four nouns, four adjectives and four verbs. In addition, all twelve words were selected from levels 1 and 2 in the vocabulary list of Japanese Language Proficiency Test (JLPT), cross-matched by JLPT levels 1–2 over three word categories of Kango, Wago and Gairaigo. As shown in Table 1, Cronbach’s alpha, calculated across 281 participants with 48 test items, was relatively high at 0.739.

**Table 1:** Full scores, means, standard deviations, and correlations for 12 language tests (observed variables).

	Language tests	1	2	3	4	5	6	7	8	9	10	11	12
**Lexical knowledge** (α = 0.739)													
1	Function words	1											
2	Japanese origin (Wago)	0.47 **	1										
3	Chinese origin (Kango)	0.44 **	0.35 **	1									
4	Loanwords	0.36 **	0.33 **	0.39 **	1								
**Grammatical knowledge** (α = 0.793)													
5	Morphological inflection	0.44 **	0.32 **	0.59 **	0.48 **	1							
6	Local dependency	0.19 **	0.16 **	0.25 **	0.23 **	0.37 **	1						
7	Structural complexity	0.44 **	0.35 **	0.51 **	0.46 **	0.68 **	0.33 **	1					
**Sentential inference** (α = 0.638)													
8	Causal relations	0.41 **	0.35 **	0.43 **	0.50 **	0.54 **	0.17 **	0.50 **	1				
9	Intentional expressions	0.32 **	0.23 **	0.40 **	0.39 **	0.51 **	0.29 **	0.39 **	0.41 **	1			
10	Co-referential resolution	0.32 **	0.29 **	0.40 **	0.35 **	0.43 **	0.10 **	0.43 **	0.39 **	0.37 **	1		
**Text comprehension** (α = 0.613)													
11	Reading comprehension	0.39 **	0.33 **	0.47 **	0.46 **	0.55 **	0.22 **	0.52 **	0.48 **	0.43 **	0.42 **	1	
12	Listening comprehension	0.35 **	0.36 **	0.42 **	0.43 **	0.44 **	0.18 **	0.40 **	0.33 **	0.34 **	0.33 **	0.46 **	1
	**Full score**	12	12	12	12	12	12	12	6	6	6	8	8
	**Mean**	4.66	3.80	6.90	7.20	8.16	10.21	7.57	3.37	4.58	2.76	4.25	4.12
	**Standard Deviation**	1.91	1.96	1.83	2.03	2.35	1.40	2.40	1.43	1.16	1.19	1.88	1.58

**Note**. N = 281

* p < .05

** p < .01

α = Cronbach’s Alpha

### Grammatical knowledge

**Fig 3:**
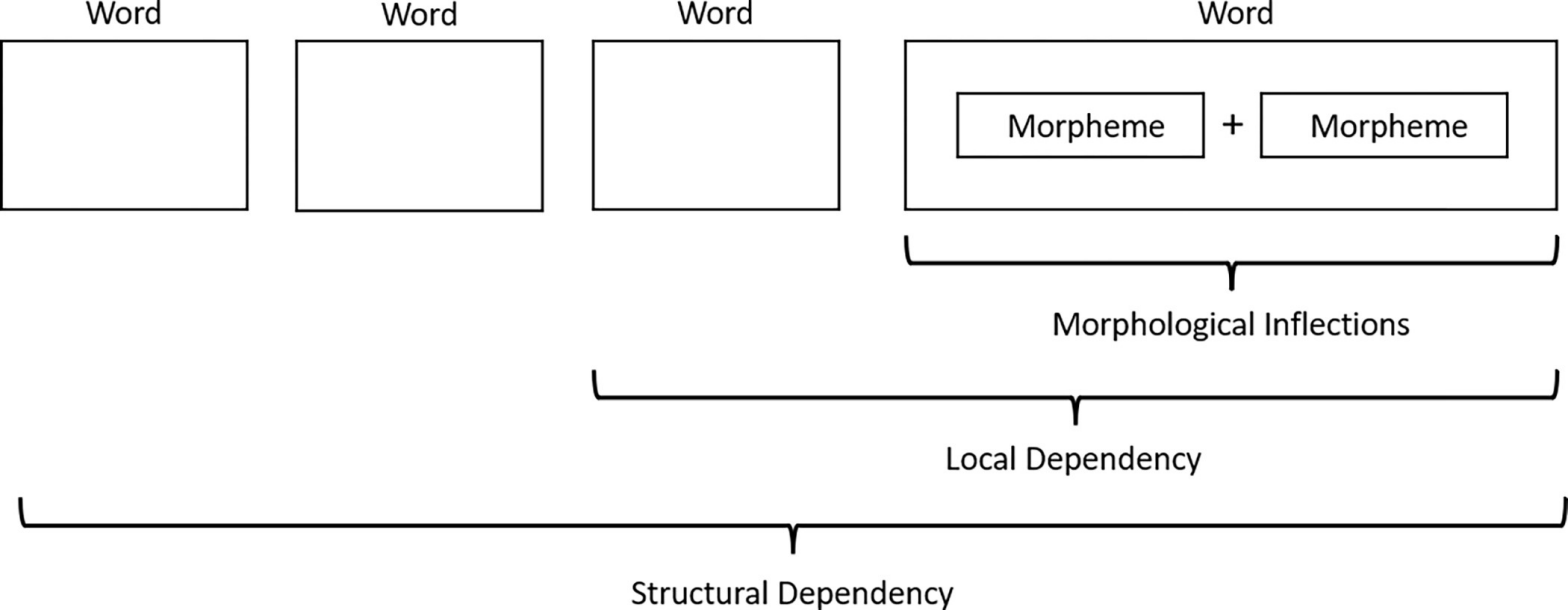
Different types of (local) dependencies in Japanese sentence comprehension.

Grammatical knowledge refers to knowledge of the rules and principles that regulate the structure and usage of language, encompassing an awareness of parts of speech, sentence structure, and the interconnectedness of words and phrases. As depicted in Fig 3, grammatical knowledge was measured through three subtests: morphological inflections, local-dependency, and complex structure. The subtest for morphological inflections consisted of questions about changes within a single lexical unit. For example, Japanese verbs and adjectives have complex inflections, and a predicate presented as a single unit in Japanese can be formed by adding various morphemic units indicating negation, past tense, voice, aspect, and mood. For instance, the verb stem *seme-ru* ‘to blame’ can be compounded with the negation–*nai* and the past tense–*ta*, resulting in the predicate of *seme-nakat-ta* ‘did not blame’. In the test, participants were asked to fill in an empty sentence bracket, such as the following example: *Ayamatte kabin-o kowasita watasi-o*, *titi-wa* () ‘My father (did not blame) me, who mistakenly broke the flower vase’. Out of four choices: *seme-nakat-ta*, *seme-nai-dat-ta*, *semeru-nakat-ta*, and *seme-naku-te-dat-ta*, *seme-nakat-ta* was the correct answer for ‘did not blame’.

As depicted in Fig 3, the subtest for local dependency required participants to determine whether two neighboring units formed a correct expression. For example, the verb *tukur-u* ‘to cook’ can be modified by the adverb *zyôzu-ni* ‘very well’ to mean ‘to cook very well.’ However, when used as an adjective to modify a noun, the particle -*na* must be used, as in *zyôzu-na e* ‘a well-drawn picture.’ The difference between these two words is that *-ni* should be used for the adverb and *-na* for the adjective. In this subtest, the correct answer for the sentence *Kanozyo-wa itumo tamagoyaki-o () tukuru* ‘She always cooks omelets (very well)’ could not be determined by lexical knowledge alone. The correct choice among *zyôzu-ni*, *zyôzu-de*, *zyôzu-no*, and *zyôzu-na* could only be determined by referencing the verb *tukuru* (to cook) at the end of the sentence.

The third subtest for complex structure required participants to refer to an entire sentence to determine the correct answer, as illustrated in Fig 3 as a long-distance relation. In the example sentence *Donnani kanozyo-ga* (), *ano daigaku-niwa gôkaku sinai darô* ‘(No matter) how hard she (tries), she cannot pass the entrance examination of that university,’ the four choices were *ganbattemo*, *ganbatte*, *ganbarunoni*, and *ganbaruga*. Each of these four expressions could be used to form a correct sentence; however, since the final phrase of the sentence ended with a negative following the prior phrase *donnani … temo* [no matter how …], the correct choice could only be *ganbattemo*. Cronbach’s coefficient alpha, calculated across 281 participants with 36 test items, was relatively high at 0.793.

#### Sentential inference

Sentential inference was measured using three subtests: intentional expressions, co-referential resolution, and causal relations. A total of eighteen questions were administered, six in each of the three categories. All questions in the three subtests consisted of more than two sentences. An example of an intentional expression is ‘Shall I fix you a coffee?’ with the response ‘If I drink coffee now, I won’t be able to sleep.’ Participants were asked to choose the best follow-up response from the first speaker: (a) ‘Then, only I will drink coffee.’, (b) ‘How much sugar would you like to have?’, (c) ‘If you take this pill, you can sleep well.’, or (d) ‘I also like coffee very much.’ The appropriate response is (a). The only cue for choosing the correct response was recognizing the intention of the listener’s statement ‘I cannot sleep.’

Coreferential resolution involves deictic words such as *kore* (this), *sore* (that), and *are* (distal ‘that’), which refer to a thing or idea from a previous sentence. For instance, in the sentence "Actually, I am afraid of cats. Please do not tell anyone about this," the phrase "this thing" (*kono koto* in Japanese) broadly refers to the speaker’s fear of cats rather than a specific word from the previous sentence.

Causal relations questions were created using multiple sentences that followed a logical progression. For example, "Last night, I read a book until 2 a.m. () it was really interesting, so I couldn’t stop reading." The correct answer was "*datte*" (because) rather than the other three choices—"*dakara*" (therefore), "*dakedo*" (even though), and "*sorede*" (then). Based on the meaning of the previous sentence, the causal expression "*datte*" explains why the speaker read a book until 2 a.m., stating that the book was captivating. These two sentences have a causal relationship, and this question evaluates understanding of causal relations. Cronbach’s coefficient alpha, calculated for 281 participants with 18 test items, was 0.638 (acceptable).

#### Text comprehension

Text comprehension is defined as the semantic understanding of a whole text’s theme. It was measured using reading and listening comprehension tests with relatively long, visually-presented (reading) or auditory-presented (listening) texts, each consisting of one or two paragraphs. There were eight texts for reading comprehension and eight for listening comprehension. Each correct answer was worth one point. To ensure that the total study workload was manageable, we included only one question per text. This approach was considered reasonable, as high-quality questions probing deeper thinking, connections, and inference generation were used, making them suitable for measuring text understanding. Cronbach’s coefficient alpha, calculated for 281 participants with 16 test items, was 0.613 (acceptable).

### Procedure

A series of tests was conducted on university students in two ninety-minute classes (180 minutes total) in quiet classrooms. Each Japanese language class had 30 students. Tests were administered to second-year students in five classes and third-year students in five classes (10 classes total) at the beginning of the academic year (September). Second-year students were considered to have one year of Japanese learning experience, while third-year students had two years. One or more of the paper’s authors and the students’ Chinese-speaking class instructors supervised the two test sessions in the 10 classes.

## Results

For the 281 participants (146 second-year students and 135 third-year students), the full scores, means, standard deviations, correlations for the twelve tests, and Cronbach’s coefficient alphas for the four latent variables are shown in Table 1.

### Model comparisons

Using AMOS [50] added on to SPSS Statistics Version 28, a SEM analysis was conducted with a covariance matrix on 281 Chinese students learning Japanese in China. The goodness-of-fit index (see Table 2) showed that the Chi-squares for both the partially-mediated model [χ2(48) = 72.25, p < .05] and the wholly-mediated model [χ2(50) = 72.88, p < .05] were significant, indicating that neither model ideally fit the data [51]. However, Chi-square tests are inherently dependent on sample size (e.g., [52–55]). Thus, Jöreskog and Sörbom [54] proposed the χ2/df index for larger sample sizes, which indicates a good fit for values less than 2.00. Since the current sample size (N = 281) was relatively large, χ2/df was calculated as shown in Table 2. This resulted in both models fitting well, but the wholly-mediated model (1.46) scored slightly lower, demonstrating a better fit than the partially-mediated model (1.51).

**Table 2:** Comparison indices of the partially-mediated model and the wholly-mediated model.

Model	χ^2^-index	χ^2^/df	AIC	CAIC	ECVI	BIC
Partially-mediated model	χ^2^ (48) = 72.25, p = 0.13	1.51	132.25	271.40	0.47	241.40
Wholly-mediated model	χ^2^ (50) = 72.88, p = 0.19	1.46	128.88	258.75	0.46	131.61
Saturated Model	--	--	156.00	517.79	0.56	439.79
Independence Model	--	--	1,268.09	1,323.75	4.53	1,311.75

*Note*. AIC = Akaike Information Criterion. CAIC = Consistent AIC. ECVI = Expected Cross Validation Index. BIC = Bayes Information Criterion.

Table 2 summarizes four model comparison measures [55], and the best-fitting model for the present data was chosen using these criteria, where a lower value indicates a better fit. As indicated by all four criteria in Table 2, the wholly-mediated model was a better fit than the partially-mediated model across all values: AIC [56], CAIC [57], ECVI [58], and BIC [59]. Consequently, the wholly-mediated model was adopted for the present study.

### The wholly-mediated model—Fit indicators

**Fig 4:**
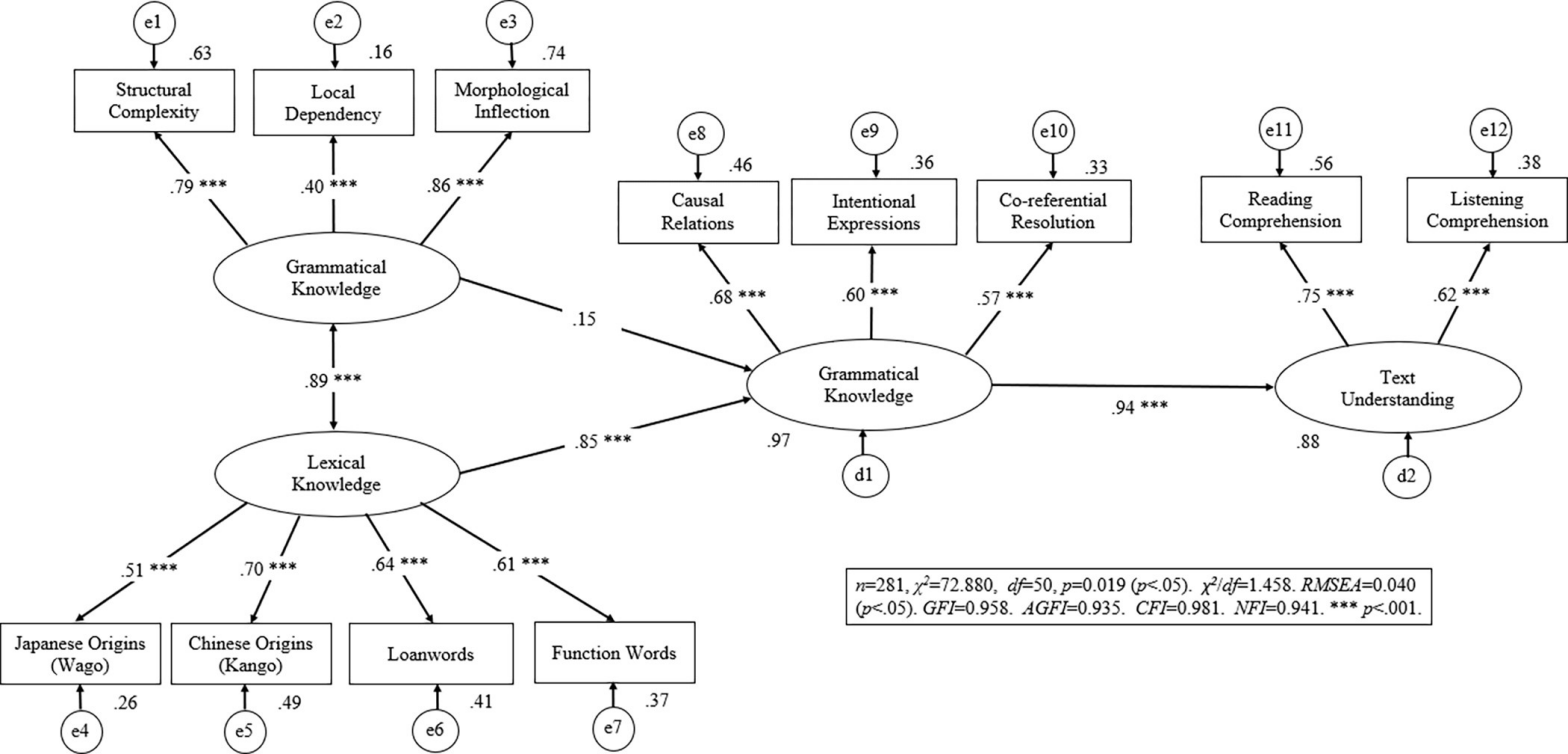
SEM results of the wholly-mediated model. Note: RMSEA: Root Mean Square Error of Approximation. GFI = Goodness of Fit Index. AGFI = Adjusted GFI. NFI = Normal Fit Index. CFI = Comparative Fit Index.

We evaluated the fit of the wholly-mediated model to the data obtained in the present study (see Fig 4) using six criteria (for details, see [53, 55]). All six indicators, namely: χ2/df [54], RMSEA [58], GFI [60], AGFI [55], NFI [61], and CFI [55] demonstrated that the model fit the data very well. Therefore, the wholly-mediated model depicted in Fig 4 was further investigated using SEM analysis.

### The wholly-mediated model–SEM results

The SEM revealed the component structure for the latent variables within the wholly-mediated model, which was constructed with four latent variables as shown by the ellipses in Fig 4. The SEM causal structure was established such that grammatical and lexical knowledge connected to text comprehension via sentential inference. These four latent variables were estimated by twelve observed variables depicted by rectangles in Fig 4. For the latent variable of lexical knowledge, all path coefficients indicated that the four observed variables contributed significantly; Japanese-origin words (beta = 0.51), Chinese-origin words (beta = 0.70), alphabetic loanwords (beta = 0.64), and function words (beta = 0.61) were all important. The latent variable of grammar knowledge was measured by three observed variables. Very high path coefficients were found for morphological inflections (beta = 0.86), and for structural complexity (beta = 0.79), but relatively lower for local dependency (beta = 0.40). Morphological inflections and, to a lesser degree, structural complexity seemed to greatly contribute to grammar knowledge.

Similarly, the latent variable of sentential inference was measured by three observed variables, which showed relatively high contributions: causal relations (beta = 0.68), intentional expressions (beta = 0.60), and co-referential resolution (beta = 0.57). The fourth latent variable of text comprehension was measured by reading comprehension (beta = 0.75) and listening comprehension (beta = 0.62), which showed a large contribution.

The wholly-mediated model was applied to investigate the influence of sentential inference on bridging the gap between lexical/grammatical knowledge and text comprehension. Since the correlation between lexical knowledge and grammatical knowledge was very high (r = 0.89, p < .001), both factors seemed to be acquired in a mutually-related manner. Native Chinese speakers displayed a strong causal relation between lexical knowledge and sentential inference (beta = 0.85, p < .001), but they showed no significant causal relation between grammatical knowledge and sentential inference (beta = 0.15, ns). Moreover, a strong significant causal relation was found between sentential inference and text comprehension (beta = 0.94, p < .001). These SEM results indicate that Chinese speakers learning Japanese rely heavily on their lexical knowledge to support their sentential inference, which in turn contributed greatly to text comprehension. In sum, a clear sequential relation from lexical knowledge via sentential inference to text comprehension was observed in the present study.

The indirect effect from lexical knowledge to text comprehension was significant (beta = 0.72, *p* < .01) while the indirect effect from grammatical knowledge to text comprehension was not significant (beta = -0.198, ns). The significant probabilities (*p*-value) of the indirect effects were calculated 500 times by bootstrapping. The indirect effects also provided evidence for the causal pathway from lexical knowledge, through sentential inference, to text comprehension in the mediated model.

## Discussion

The primary goal of reading or listening to a text is to grasp the intended message. While lexical knowledge and basic grammatical understanding may be sufficient for comprehending individual sentences, it often falls short when trying to understand a series of sentences in Japanese or other languages. This study aimed to explore the role of sentential inference in bridging the gap between lexical/grammatical knowledge and overall text comprehension. Four tests (i.e., lexical knowledge, grammatical knowledge, sentential inference, and text comprehension) were conducted on 281 native Chinese-speaking students learning Japanese at a Chinese university to examine the causal relationships among these abilities. Two distinct models were considered: the *partially-mediated model*, which views sentential inference as just one of many factors contributing to text comprehension on par with lexical/grammatical knowledge; and the *wholly-mediated model*, which posits sentential inference as separate from lexical/grammatical knowledge and serving as a bridge to broader text comprehension (e.g., [10–14]). In this model, sentential inference is crucial for integrating sentence-level information to understand the entire message of a text. Our SEM analysis revealed that the wholly-mediated model better fits the data than the partially-mediated model, as further demonstrated in Fig 4. This finding suggests a sequential causal relationship that starts with lexical knowledge (but not grammatical knowledge), moves through sentential inference, and ultimately leads to text comprehension. The current study highlights the importance of sentential inference as a central mediator of lexical knowledge in facilitating holistic text understanding in Japanese.

In this study, we analyzed participants who had completed one and two years of Japanese language learning together. However, considering that increasing proficiency could potentially affect the variables being investigated, we re-evaluated the data using the wholly-mediated model for Grade 1 (N = 146) and Grade 2 (N = 135) separately. This analysis produced results like those obtained when combining all participants (N = 281). Nevertheless, the goodness-of-fit indices were lower than those for the full sample. Consequently, we opted to retain the single-group structural equation modeling (SEM) analysis with a sample size of N = 281.

The core finding of this study is that sentential inference is essential for bridging the gap between lexical knowledge and comprehensive understanding of Japanese texts. In a study on learning Chinese as a foreign language, Zhang et al. [29] discovered that lexical inference served as a connecting tool between morphological awareness/lexical knowledge and Chinese reading comprehension for native English speakers. As Chinese texts are entirely written in hànzì, English speakers must first acquire knowledge of the morphological units of hànzì characters to read Chinese. Lexical inference is automatically employed as a mediating factor for reading comprehension in Chinese. Consequently, differences may exist in how Japanese and Chinese text comprehension occurs. Specifically, sentential inference might act as a bridge between lexical knowledge and Japanese text comprehension, while lexical inference appears more involved in understanding Chinese texts. It should be noted that it is also possible that sentential inference connects Chinese vocabulary knowledge and text comprehension, with the only difference being that lexical inference is required prior to sentential inference (note: we like to thank an anonymous reviewer for pointing this out).

Although not examined in our current paper, it is conceivable that individual differences in working memory capacity also contribute to complete text understanding. For instance, successful inferences must be stored in memory, particularly for long and dense texts, as subsequent interpretation relies on them. Future research is needed to determine if this factor is significant for our empirical setup.

It is important to note that our findings are currently interpreted in the context of successful and complete text comprehension. Our tests assessed whether whole text understanding was achieved, and our model was built on this data. This does not imply that every reading situation must follow this exact pattern (e.g., not all text comprehension results in complete understanding). Undoubtedly, scenarios may exist where, for example, a low-proficiency L2-learner could still benefit from lexical information aiding text comprehension despite not fully achieving sentential inference. Other cases might involve native speakers confronted with complex legal texts, which typically demand significant inferencing skills from readers. However, failure to achieve sentential inference often leads to incomplete overall comprehension, causing nuances or even essential points to be misunderstood.

Considering the findings from the current study, several implications for L2 acquisition and teaching could be drawn. First, the results underscore the importance of emphasizing lexical knowledge development in language learning, as it appears to play a more significant role in sentential inference and text comprehension than grammatical knowledge. Consequently, L2-educators might consider prioritizing the development of students’ vocabulary, focusing on both the breadth and depth of their lexical knowledge. Second, the study highlights the need to focus on sentential inference training in L2-instruction. Educators might therefore incorporate activities and strategies that enhance students’ abilities to make inferences at the sentence level, such as tasks that involve identifying context clues, drawing connections between words, and predicting the meaning of unfamiliar words or phrases. Lastly, our findings potentially suggest a reevaluation of the role of grammar instruction in L2-acquisition and teaching. Given that grammatical knowledge seems not to significantly contribute to text comprehension through sentential inference, L2-educators may need to place greater emphasis on communicative competence and contextual understanding, though further research, admittingly, needs to explore the role of grammar in different aspects of language learning and proficiency.
