# Correction: Sentential inference bridging between lexical/grammatical knowledge and text comprehension among native Chinese speakers learning Japanese

**DOI:** 10.1371/journal.pone.0331311

**Published:** 2025-08-28

**Authors:** Katsuo Tamaoka, Hiromu Sakai, Yayoi Miyaoka, Hajime Ono, Michiko Fukuda, Yuxin Wu, Rinus G. Verdonschot

In Fig 4, the middle label Grammatical Knowledge is incorrect. The correct label is Sentence Inference. Please see the correct Fig 4 here.

**Fig 4:**
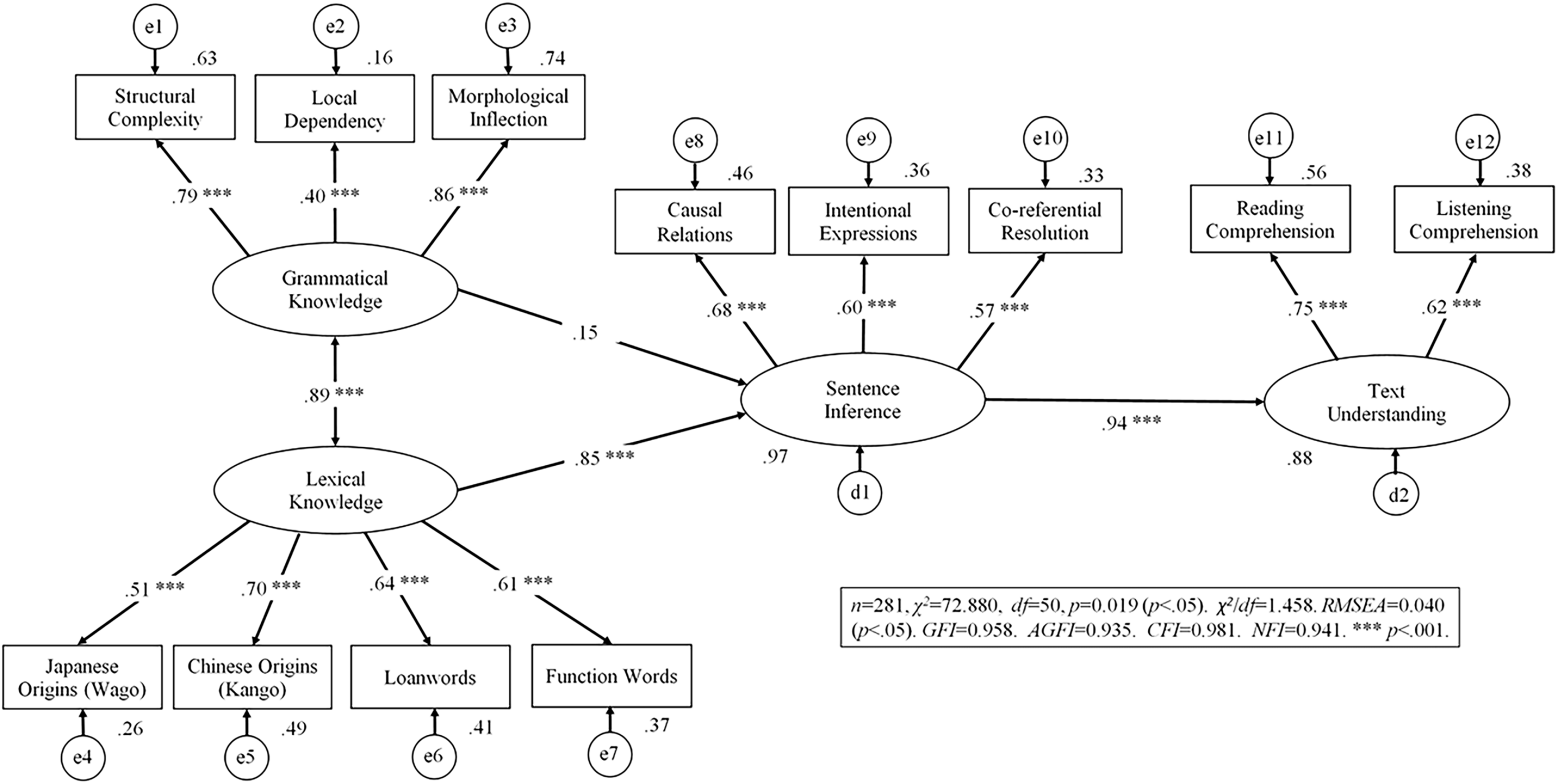
SEM results of the wholly-mediated model. Note: RMSEA: Root Mean Square Error of Approximation. GFI = Goodness of Fit Index. AGFI = Adjusted GFI. NFI = Normal Fit Index. CFI = Comparative Fit Index.
